# Self-care management and experiences of using telemonitoring as support when living with hypertension or heart failure: A descriptive qualitative study

**DOI:** 10.1016/j.ijnsa.2023.100149

**Published:** 2023-08-05

**Authors:** Susanna Strandberg, Sofia Backåberg, Cecilia Fagerström, Mirjam Ekstedt

**Affiliations:** a Department of Health and Caring Sciences, Linnaeus University, Universitetsplatsen 1, Kalmar, Växjö 351 95, Sweden; b Faculty of Kinesiology, University of Calgary, Calgary, Canada; c Department of Research, Region Kalmar County, Kalmar, Sweden; d Department of Learning, Informatics, Management and Ethics, Karolinska Institutet, Stockholm, Sweden

**Keywords:** Chronic disease, eHealth, Informal caregivers, Patient, Primary care, Qualitative research, Self-care, Telehealth

## Abstract

**Background:**

The need for support in self-care at home will increase with the growing older population with chronic illness. Many people have one or more chronic illnesses and struggle with self-care activities, often supported by informal carers at home. The rapid development of telemonitoring applications in primary care calls for increased knowledge about how people with chronic illness and their informal carers experience the use of telemonitoring applications at home.

**Objective:**

This study aims to describe experiences of self-care management at home when living with hypertension or heart failure, with support from primary care through telemonitoring.

**Design:**

A descriptive qualitative approach was applied using semi-structured interviews with patients and informal carers in a pilot project on telemonitoring of chronic illness in primary care from October 2019 to June 2021.

**Setting:**

Participants were recruited from three primary care settings and one medical department at one hospital in a region in southern Sweden.

**Participants:**

A purposive sample of patients (*n* = 20) with chronic illness living at home and their informal carers (*n* = 4) were recruited.

**Methods:**

Semi-structured telephone interviews were conducted, guided by open-ended questions targeting patients’ and informal carers’ experiences of self-care management at home and using telemonitoring applications as support. Transcribed interviews were analyzed through qualitative content analysis.

**Results:**

‘Developing the capability to perform self-care with technology as both an intruder and an invited guest’ was the unifying theme that tied together the experiences of patients with chronic illness and their informal carers. Experiences of self-care management included acquiring necessary self-care skills, expertise in managing their chronic illness, and reciprocal relationships with healthcare professionals when using telemonitoring application as support in self-care monitoring of vital parameters. However, uncertainty regarding the interpretation of symptoms and a feeling of exclusion were seen.

**Conclusions:**

Telemonitoring applications offer potential support for patients with chronic illnesses and their informal carers, enabling them to establish new routines and enhance motivation for self-care activities at home. This study emphasizes the adaptability of telemonitoring applications in meeting the unique support requirements of patients and informal carers when managing self-care at home.

## Contribution of the paper

What is already known

• The number of people with chronic illness is growing worldwide, and many of them and their informal carers manage self-care at home.
• eHealth solutions like telemonitoring applications can support people with chronic illness and their informal carers to engage in self-care at home.
• Knowledge regarding how telemonitoring applications can be used when engaging in self-care at home needs to be developed to improve dissemination and uptake in primary care.

What this paper adds

• Patients with hypertension or heart failure and their informal carers may gain knowledge about their illness and improve their ability to adapt self-care management by using a telemonitoring application.
• Use of telemonitoring applications at home may enhance both confidence and motivation for self-care activities through tailored feedback.
• Utilization of telemonitoring applications facilitates establishment of new routines and health objectives in daily life by fostering a reciprocal relationship between patients and healthcare professionals.

## 1 Background

Chronic illnesses, commonly referred to as ‘non-communicable diseases’, currently constitute the primary cause of premature death worldwide. According to the World Health Organization, non-communicable diseases encompass five distinct chronic conditions: cardiovascular diseases, stroke, cancers, chronic respiratory diseases, and diabetes. Cardiovascular diseases are among the most common (World Health Organization, 2021). Chronic illnesses are associated with medical complexities such as polypharmacy or functional disabilities, increased utilization of care and mortality (Dent et al., 2019; Kuipers et al., 2020). Living with a chronic illness affects a person's quality of life by intruding on functional ability, the ability to work and activities in daily life (Ambrosio et al., 2015; Violan et al., 2014). With older age, the risk of chronic illness increases (Barnett et al., 2012). Given the growing older population worldwide, there is a rising need for self-care activities for the prevention and management of chronic illness in the future (World Health Organization, 2021).

Living with chronic illness at home requires high self-management capacity, where the knowledge, skills, and competence for self-care are essential (Riegel et al., 2019; 2012). Self-care includes a process of health maintenance, monitoring for changes in signs and symptoms, and management of those changes when they occur (Riegel et al., 2019). This involves learning health-promoting behaviours, such as adopting a diet, engaging in regular physical activity, monitoring blood pressure, and adhering to medication regimens, which may contribute to maintaining health and managing illness. It also includes building the capacity and motivation to perform these behaviours consistently over time. Self-care includes monitoring, which is a process of surveillance or body listening, for example monitoring blood pressure or weight with a focus on recognizing any changes that occur (Matarese et al., 2018). Bodily changes can be signs or symptoms related to the chronic illness and may be grounds for a decision on what actions should be taken regarding self-care management and communication with healthcare services (Riegel et al., 2019; 2012).

Multiple factors contribute to the difficulties faced by individuals with chronic illnesses. Many struggle with steady deterioration of health and dependency on informal carers or face barriers when interaction is needed with healthcare professionals (Boehmer et al., 2016; Ploeg et al., 2017). In a study following older individuals with heart failure for one year after hospitalization, it was observed that most of them faced difficulties in carrying out essential tasks associated with self-care for heart failure. Interestingly, there was a weak correlation between self-perceived ability and actual performance, and individuals with poor self-care abilities were found to have a higher risk of mortality within one year (Vidán et al., 2019).

Thus, managing illness and health-promoting practices at home is often challenging, and not everyone is capable of performing self-care at home without adequate support (Riegel et al., 2019, 2012). Support with self-care monitoring from people in the home environment, such as informal carers, can influence and increase self-care competence among people affected by chronic illness or functional disability (Matarese et al., 2018; Vellone et al., 2019). An informal carer could be a family member, friend or neighbour who provides unpaid help to a person with a chronic illness on an irregular basis (Eurocarers, 2020). Informal carers are often involved in managing the chronic illness and therefore also need knowledge and support to manage self-care at home (Vellone et al., 2015).

The care needs of people with chronic illness should be addressed through person-centred care, which is difficult in today's fragmented healthcare system (Wallace et al., 2015). The development of person-centred care including health-promoting environments is essential to improve prevention and reduce risk factors for chronic illnesses (Holland and Lee, 2019; World Health Organization, 2013). This is particularly significant for primary care, which is the first line of healthcare, offering treatment and follow-up of patients with chronic illnesses (Moody et al., 2022; Violan et al., 2014).

Implementation of eHealth solutions has increased the potential of primary care to offer person-centred support and meet patients’ specific self-care needs (Hanlon et al., 2017). Self-care monitoring through telemonitoring technology gives people with chronic illness and their informal carers access to healthcare services at a distance (Hanlon et al., 2017; Lefler et al., 2018). For example, the use of telemonitoring applications enables for healthcare professionals and the patient to track the patient's vital signs and health over time, which can reduce hospitalization rates (Gjestsen et al., 2018; Li et al., 2021). Few studies have focused on supporting people with chronic illness in self-care monitoring, including bodily signs and symptom management (Buck et al., 2018; Riegel et al., 2021). The significance of informal carers in the management of chronic illnesses is attracting growing attention, particularly as interventions are being broadened to encompass informal carers (Buck et al., 2018; Vellone et al., 2019).

It has been found that patients with chronic illness can use telemonitoring applications, especially if healthcare professionals are available when needed, although they often receive support in such use from informal carers. There are patients with chronic illness who, through the use of telemonitoring applications, take a more active role in self-care monitoring and gain a better understanding of their symptoms and care (Aamodt et al., 2020; Hanley et al., 2018). For self-care interventions to be successful, they should be based on a holistic approach to enhance the patient's capacity to sustain their physical and cognitive functioning, their independence and their overall well-being for an extended period (Riegel et al., 2022a). There is still only limited knowledge on how high-quality care that supports self-care management can be delivered (Dent et al., 2019). There is a need for further studies focusing on the experiences of self-care management and health promotion among patients with chronic illness using telemonitoring in primary care (Bajraktari et al., 2020). This study aims to describe experiences of self-care management at home when living with hypertension or heart failure, with support from primary care through telemonitoring.

## 2 Methods

### 2.1 Study design and setting

This study has a descriptive, qualitative approach using individual semi-structured interviews. An inductive qualitative analysis, including manifest and latent content at different levels of interpretation (Elo and Kyngas, 2008; Lindgren et al., 2020), was considered appropriate to provide a comprehensive understanding of the patients’ and informal carers' experiences of self-care management at home when living with chronic illness, with support from primary care through telemonitoring.

The study was conducted in a primary healthcare context in a region in southern Sweden as part of a pilot project focusing on continuity, safety and participation in self-care for patients with various chronic illnesses. Patients with chronic hypertension or heart failure got access to a telemonitoring application for self-care monitoring of vital signs and symptoms during 6 months. Two different telemonitoring applications were used at three primary healthcare centres and a medical department at one hospital from October 2019 to June 2021. Both telemonitoring application for smartphones or tablets included chat functions, self-monitoring of blood pressure, bodyweight, heart rate, oxygen saturation and body temperature, and a questionnaire for reflecting on vital symptoms and health status. Differences between the two applications were that one offered an additional feature of video calls, which required access to power via an electric socket, limiting the locations where it could be used. The other telemonitoring application, by contrast, could be installed on a patient's mobile phone. The data were digitally transferred to the primary healthcare centres or the medical department. Nurses monitored health data through the application Monday to Friday and could contact the patients through the video or chat function. Patients were contacted for consultations and self-care guidance only if vital signs deviating from the normal range were recorded or when patients themselves made contact due to questions that had arisen. The differences between the two telemonitoring applications were not crucial for patients’ daily monitoring or regular use; therefore, we did not consider these differences in this study.

### 2.2 Participants and data collection

Purposive sampling was used to get the most heterogeneous sample and diverse experiences possible (Polit and Beck, 2012). Inclusion criteria for participants in this study were: having one of the chronic illnesses hypertension or heart failure as a main diagnosis, being part of the pilot project and having tested the telemonitoring application for six months, independently or with an informal carer living at home. Nurses working with telemonitoring applications at the primary healthcare centres or the medical department recruited eligible patients and informed them about the study through verbal and written information. The first author then contacted each participant who had accepted the invitation by telephone, for further information and setting up a time for an individual interview. During the interview process, participants were asked if they had an informal carer who was involved in the use of the telemonitoring application, and if they consented to their informal carer being asked to participate. Four informal carers were then invited to participate in individual interviews. The inclusion criteria for the informal carers were living with a person with hypertension or heart failure and having taken part in the use of the telemonitoring application. Exclusion criteria were not speaking Swedish or English. No age limit was used. Out of a total of 23 patients asked to participate in the study, 20 chose to do so; see Table 1. Three did not respond and did not explain why they did not participate.

**Table 1:** Overview of demographic information.

Patients	*n* = 20
Age in years, Md (min–max)	72.5 (50–88)
Sex, female	9
Diagnosis *Heart failure* *Hypertension*	13 7

Informal carers	*n* = 4
Age in years, Md (min–max) Sex, female	75 (71–77) 4

Data were collected between March and November 2020 and all interviews were conducted by the first author. Due to the COVID-19 pandemic, the interviews were held by telephone. The interviews were semi-structured, based on an interview guide and lasted 30–75 min. The interview guide was organized with sets of open-ended questions covering specific areas of the aim (Appendix A)*.* Kvale et al. (2014) consider knowledge to be created through lived experiences – the interactions between a person and the world. During the interviews, the focus was on seeking nuances in how use of a telemonitoring application was perceived as part of self-care through lived experiences (Kvale et al., 2014). We obtained the participants' consent to re-establish contact in the event of any uncertainties from the interviews requiring further explanation.

### 2.3 Data analysis

An inductive qualitative approach was used to identify patterns of similarities and differences in experiences of using telemonitoring as part of self-care at home. The inductive approach is data-driven and can have various levels of abstraction and interpretation in the search for patterns (Graneheim et al., 2017; Graneheim and Lundman, 2004). Qualitative content analysis is a systematic method that offers an opportunity to analyse data at both a manifest and a latent level, where categories represent the manifest level and themes represent the latent, underlying meaning (Graneheim and Lundman, 2004; Lindgren et al., 2020).

The interviews were audio-recorded and transcribed verbatim, with transcripts constituting the unit of analysis. The transcripts were read by the authors several times while listening to the recordings, to gain a sense of the whole. Participants' names and other identifying data were removed from the transcripts. The qualitative data analysis software NVivo (QSR International Pty Ltd., 2020) was used to manually sort and structure the data. Sentences related to the aim of the study were identified as meaning units. Thereafter, the meaning units were condensed and abstracted into codes and labelled (Graneheim and Lundman, 2004). Codes with similar contents were sorted into sub-categories and categories were thereafter identified jointly by all authors. During the analysis, the authors moved between closeness and distance when codes and subcategories were compared and grouped, which indicates that different levels of abstraction were used (Lindgren et al., 2020). All authors took part in the abstraction and interpretation process leading to subcategories, categories and an overall theme. An example of the analysis process is described in Table 2. Reflections and discussions between all authors were conducted during the process to achieve greater understanding and consistency in the analysis process and to avoid pre-understandings affecting the analysis. The analysis resulted in three categories, eight subcategories and one main theme, running through the categories as an interpretation of the latent meaning (Graneheim et al., 2017).

**Table 2:** Examples of the analysis process.

Meaning unit	Condensed meaning unit	Code	Subcategory	Category
No, that aspect is something we haven't … it hasn't even been discussed, that anyone has a goal with, like … // No, I'm afraid no one has any goals, it's just making sure that you survive until the next doctor's visit, basically.	Never having had goals with healthcare, simply trying to survive between care visits	A lack of goals	Being left to rely on a digital tool	Reciprocal relationship with healthcare professionals
But that would be something, like when I was talking to the healthcare centre … It was what they said at the start, that if they saw anything, then they would contact the hospital, I think?	A lack of coordinated care between caregivers and rely on that healthcare has a care plan	Rely on that healthcare has a care plan		
Yeah, I just use this page and write in that case, but I think that the message box is great to have, because then I know that I will be contacted if I write something there, then I know that I'll be contacted after that.	Writing in the chat to get in contact with healthcare quickly	Simpler communication pathways	Developing relationships through a digital tool	
Yeah, the thing is that some doctor has looked at my values and she thought that … no, this isn't good, your heart rate is too high, so you should probably increase your dose of your medicine.	Pharmaceutical adjustments based on deviating values after assessment by healthcare	Joint creation of a pharmaceutical treatment plan		

### 2.4 Trustworthiness

The trustworthiness of qualitative studies relates to credibility, dependability, transferability, and confirmability (Graneheim et al., 2017; Graneheim and Lundman, 2004). A purposive sampling was used to obtain a broad sample and achieve credibility in describing the patients’ and informal carers’ experiences of self-care when using a telemonitoring application. A purposive sample was used to find people who had experiences of the phenomenon being studied and because participants needed to be recruited from a specific organization (Polit and Beck, 2012). The decision to include informal carers in the data collection was made to gain a greater understanding of the phenomenon and better address the aim of the study. Moreover, to achieve credibility, the data analysis processes were underpinned with detailed examples showing that the data included rich variations. Also, to avoid the analysis being affected by pre-understandings and to achieve dependability and confirmability, the authors jointly discussed and reflected on the codes and categories. Quotations are presented to enhance authenticity, achieve confirmability and reveal nuances in the experiences. In addition, the quotations can be evaluated by readers to determine if the results are transferable to similar contexts. Information power is intrinsically associated with the particularity of experiences, knowledge, or attributes exhibited by participants. The individual encounters with participants – both patients and informal carers – combined with the possibility to discuss matters over the telephone, the aim of the study, and the thorough analysis, resulted in high information power (Malterud et al., 2016).

### 2.5 Ethical considerations

The World Medical Association's ethical guidelines were applied to this study (World Medical Association, 2013). All participants received verbal and written information about the aim of the study and all participation was voluntary and could be withdrawn from the study at any time. All participants gave their consent to participate in the study. The study was approved by the Ethical Review Board (DNR: 2020–01,219 and 2019–00,889).

## 3 Results

The main theme – the unifying thread that was found in data when searching for the meaning in the participants’ experiences of self-care with support from primary care through telemonitoring – was: ‘*developing a capability to perform self-care with technology as both an intruder and an invited guest*’. Over time, patients and informal carers gather experiences of living with a chronic illness and use these experiences to adjust activities and routines in everyday life. The patients could feel both excluded and included through the use of the telemonitoring application, like strangers in their own homes. Often, someone in the patient's home, an informal carer, assisted in the use of the telemonitoring application, and the application was not tailored to the patient's everyday life. When they did not master the monitoring technology, they felt like they were being thrown out into the world of technology, a different reality than what they were used to. Telemonitoring also created a new type of closeness with healthcare professionals and enhanced these relationships. The application enabled patients to feel safe at home, but the handling of technical devices could also entail a feeling of insecurity. The results are presented in Table 3. The quotes are labelled with ‘patient’ or ‘informal carer’, followed by a participant number.

**Table 3:** An overview of the theme, categories, and subcategories presented in the results of the study.

Theme	Developing a capability to perform self-care with technology as both an intruder and an invited guest		
CATEGORY	Acquiring necessary self-care skills	Reciprocal relationship with healthcare professionals	Developing expertise in managing a chronic illness
SUBCATEGORY	Knowing what to do through lived experiences	Being left to rely on a digital tool	Integrating new routines into daily life
	Getting the necessary information and support for self-care	Developing relationships through a digital tool	Developing motivation through instant and tailored feedback
	Confidence to adapt self-care activities		Uncertainty in monitoring and interpreting bodily expressions

### 3.1 Acquiring necessary self-care skills

A person living with a chronic illness acquires the necessary self-care skills to manage self-care of their illness over time. Through lived experiences and receiving necessary information and support, including the use of telemonitoring applications, they gain confidence in adapting self-care activities at home.

#### 3.1.1 Knowing what to do through lived experiences

The patients described deterioration in their chronic illness over time, with some noticing this faster than others in everyday life. Having a chronic illness requires acceptance that the illness will last for the rest of one's life and not being afraid of being acutely impaired. Those affected need to accept changes due to the chronic illness in addition to the bodily changes that occur over time and with age. Not being able to cope with everyday life in the same way as before, having increased fatigue*,* or wanting to do more than the body can manage were perceived as functional deteriorations that had to be dealt with. Patients described that they had more symptoms during some periods, such as fatigue, difficulties sleeping, dry mouth or weight gain, all of which led to decreased strength in everyday life. Limitations resulting from the chronic illness were described as affecting patients, for example during daily walks, meaning that walking distances grew shorter.

The patients reported that living with a chronic illness had given them more understanding of the symptoms that could arise and how their bodies reacted. They learned to recognize when their body was in imbalance as well as what could trigger increased symptoms, for example drinking coffee leading to an increased heart rate. Another example was that pain in the heart area meant that rest was needed.

*Yeah, I don't have that much, but I do know that I've had it for a long time, I've had it since ’90 or so, so I've had it for about 30 years or so, so I guess you learn a lot about yourself. (Patient 1)*

Information about the illness was given during hospitalizations. During normal periods, most patients did not feel sick, despite their chronic illness. Informal carers described being familiar with signs of deterioration and thus being able to draw the patient's attention to the symptoms. The patients were aware of the drug treatment necessary to prevent or avoid worsening symptoms. When the illness deteriorated and symptoms grew more prominent, the patients understood that their health would decline in the future.

#### 3.1.2 Getting the necessary information and support for self-care

Patients exemplified support from healthcare professionals about self-care as information regarding exercise's positive effect on chronic illnesses, or recommendations on diet, fluid restrictions and drug treatment. The patients also described support from homecare services with some of their self-care activities, such as leg dressings.

*I've gotten good advice and I've been there a few times. I've had … and then also when it comes to medication here, I'm taking quite a lot now. But I feel that I've gotten good support and advice from healthcare. (Patient 20)*

Patients also mentioned requiring knowledge on how to contact healthcare services when symptoms or deterioration arose, to get support on adjustments of self-care activities such as drug treatments. The patients had one or more contact routes to the healthcare services depending on the situation that had arisen and what level of care was needed. Most patients had a contact nurse at a clinic or health centre as a result of their chronic illness, but could also seek healthcare through telephone support, an emergency room or medical clinics. Informal carers and patients said that they helped each other with everyday household tasks at home, such as cooking and cleaning. Informal carers supported their sick relatives by preparing an adapted diet based on dietary advice for the illness, helping with support stockings or being available as support during exercise.

*..but I'm nearby and when he's taking a shower and things like that.. like in the bathroom and so on.. I'm always nearby just in case he gets a drop in blood pressure, which he sometimes does. (Informal carer 1)*

Both patients and informal carers said that they gave each other motivation by performing activities together. However, it also emerged that sometimes both parties were ill, which made it difficult for them to support each other in everyday life and maintain self-care.

#### 3.1.3 Confidence to adapt self-care activities

The patients described themselves as being able to avoid deterioration in their chronic illness, for example by performing self-care activities when symptoms arose. Some had prior to telemonitoring developed the confidence to independently adjust drug treatment to deal with arising symptoms, although they knew about the side effects that could arise later on.

*Then I'll wake up with pain in my chest and I'll get up and then the cramp (angina pectoris) comes, so I'll take some of the spray and it disappears in two or three minutes. So, yeah … that doesn't really worry me, I guess. I am not worried about this. (Patient 6)*

Adapting self-care activities in everyday life to their level of ability was described by the patients as an opportunity to perform the self-care that was needed. For example, some had started biking, because walking was no longer an option or had cut down to part-time work due to fatigue or other symptoms. Assistive devices, such as a walker to support balance, a heart rate sensor to monitor heartbeat or a cell phone alarm as a reminder to take medication at the right time, could be used to maintain self-care activities and adapt to the chronic illness. Living in the present, performing everyday activities and not thinking too much about how the chronic illness might affect one in the future was described as a strategy to avoid deterioration. Other strategies were staying optimistic and taking responsibility for one's health, for example by writing a diary or sticking to a healthy diet.

*Yeah, you want to hang around a while. You want to hang around a while and see your grandkids grow up and so on, so that's why you want to stay active and keep moving and so on. (Patient 4)*

Motivation was described as essential for achieving the goals of self-care activities to extend life. Received support from family and friends to continue performing self-care and having good relationships with them were considered to improve motivation. Also, positive outcomes (i.e., feedback) from performing self-care activities were described as motivating – for example, patients noticed that their breathing improved.

### 3.2 Reciprocal relationship with healthcare professionals

Patients described sometimes feeling excluded and insecure during interactions with healthcare professionals, leaving them to rely on the digital tool for support at home. On the other hand, the digital tool presented an opportunity to build reciprocal relationships between patients, informal carers, and healthcare professionals.

#### 3.2.1 Being left to rely on a digital tool

Patients sometimes felt excluded from management of self-care and sensed a lack of responsibility in contact with healthcare professionals. The patients did not always consider communication to be necessary in the event of worsening of their chronic illness, and waited until symptoms became more acute before contacting healthcare services. They thought instructions from healthcare professionals were sometimes too difficult and became meaningless. The patients also described a lack of goals and care plans regarding treatment and self-care activities and said that the annual follow-up did not give the information or the opportunity to set goals to avoid deterioration in their chronic illness.

*That stuff about goals and follow-up and so on, that doesn't exist. I don't think so, at least. I mean, it's chronic. It is chronic, they're chronic, both of what I have, and it has to be, crassly – it's chronic. (Patient 5)*

The patients described difficulties in maintaining self-care activities due to multiple specialists being involved in their healthcare. A lack of follow-ups of self-care activities or a lack of contact with healthcare professionals was experienced as a difficulty by the patients. The patients reported that motivation to perform self-care was lacking and that motivation was lost when improvements in bodily symptoms did not occur. They stated that there was no difference when the telemonitoring application was used in the contact between the patients and the healthcare centre and that the application did not create any motivation or support for self-care. Continuing with the telemonitoring application after six months was not seen as meaningful.

#### 3.2.2 Developing relationships through a digital tool

The telemonitoring application was experienced as a new, useful, faster and easier contact route with healthcare services. Communication took place online, so physical meetings could be avoided, and healthcare became more accessible according to the patients. The patients stated that the healthcare professionals seemed engaged when contact was made through the telemonitoring application. Questions that arose were answered promptly and effectively and the conversations that followed were perceived as supportive. Patients further described that using the telemonitoring application increased their participation in care, attention, support, and trust in healthcare professionals. Furthermore, the patients felt that contact through the application became more personalized, more extended and more adapted to their unique care needs as a result of reporting measurements.

*Yeah, you are (involved), I am. It feels like you are … part of the team, I guess you could say. (Patient 14)*

The patients and informal carers described setting target vital signs in the telemonitoring application together with the healthcare professionals. Enhanced support and adjustment from the healthcare professionals related to the achievement of target vital signs was experienced by the patients. Adjustment of the frequency with which measurements should be reported could be made as needed, which the patients saw as positive. Moreover, the patients stated that measuring and seeing vital signs daily created a sense of security and they appreciated that healthcare got an overview of reported vital signs and therefore could adapt care based on changes in vital signs. Both patients and informal carers felt that they could maintain self-care activities better when they were in control of the measurements taken in the telemonitoring application.

*Since I've started with this, I haven't had to go to the ER or contact anyone other than … than the heart failure nurse. I feel like it's a layer of security, having that option. (Patient 8)*

The patients also described feeling safer with drug treatment than before, due to the feedback and communication with the healthcare professionals through the telemonitoring application. Measurements reported via the telemonitoring application and contact with healthcare professionals could lead to drug adjustments, which the patients felt created a sense of security.

### 3.3 Developing expertise in managing a chronic illness

In order to develop expertise in managing a chronic illness, it is essential to integrate new routines into daily life. The utilization of a digital tool to monitor symptoms and bodily signs of the chronic illness might introduce uncertainties. However, the instant and tailored feedback through the digital tool created motivation for patients to perform self-care activities at home.

#### 3.3.1 Integrating new routines into daily life

The patients stated that behavior had changed with the use of the application, with them contacting healthcare before deterioration or increased symptoms occurred due to their chronic illness. New routines for self-care were created with the telemonitoring application based on when during the day vital signs were to be reported. Some patients reported occasionally having delayed their breakfast as they prioritized reporting vital signs through the telemonitoring application. They compared the new routine of reporting vital signs with brushing one's teeth in the morning. At the end of the trial period, the patients were amazed that an everyday habit had been established.

*Yeah, now it would be, to my mind, a disaster if that were to stop. I'm so used to these routines and can see that they help me, so that … so that … I'd prefer it to continue. (Patient 8)*

The patients described that the use of the telemonitoring application meant that measurements were more regulated and vital signs were more stable. The telemonitoring application and the equipment were described as easy to use to perform self-assessments in everyday life. The patients said that taking measurements every day with the telemonitoring application was experienced as equivalent to receiving a medical examination at home. The patients and informal carers said that the telemonitoring application provided information on the patient's health and the effects of the self-care activities that were carried out. The new routine was thought of as creating new knowledge about the patient's health status. They also experienced increased responsibility and were impressed by themselves that they have managed to use the telemonitoring application and associated equipment. Further, the patients described the telemonitoring application as serving as a reminder to maintain self-care during the trial period.

##### 3.3.2 Developing motivation through instant and tailored feedback

Getting an overview of vital signs and being able to go back and see development or stability over time was described as interesting. There were patients whose informal carers provided support in using the application by participating in the sampling, answering questions about their feelings or taking measurements. It was said that the vital signs were objective and could be shown to healthcare professionals at a healthcare centre. Getting feedback on self-care activities increased the motivation to continue using the telemonitoring application, increase physical activity and get more steps per day, for example. The motivation to improve their vital signs was increased if patients could see and follow the vital signs in the telemonitoring application. They described that having a feeling of a competition against themselves or that ‘Big Brother sees you’ led to increased motivation to improve vital signs and maintain self-care. The patients described that the informal carers' technical competence was an asset that helped them use the application correctly, for example during chats or video calls with healthcare professionals. Informal carers could also on occasions explain health information provided by the telemonitoring application that patients had not understood.

*Yeah, but, it's been like… I've gotten help with that information too, you know. I don't understand all the words, so that's like … my wife has to help me with that. She is everything, you know. Yeah, that's… I have trouble coming up with any examples, to be honest (laughing). It might be something that I don't really understand, that she can understand better, and then she'll explain it to me. (Patient 13)*

Patients who used the application together with an informal carer got peer support that might have enhanced their motivation to sustain self-monitoring and self-care activities. They also had access to feedback and interactions with healthcare professionals, which further improved their expertise for self-care. Informal carers participated in the use of the telemonitoring application and thus got an overview of measurements. Some informal carers experienced that increased involvement had been created thanks to the possibility in the application for both partners in a couple to participate in contact with healthcare.

*Yeah, but they have cheered me on here (in the app), that the values look good today and we're glad it went so well and … yeah, they (the healthcare professionals) have written a lot here, they really have.* (Informal carer 2)

##### 3.3.3 Uncertainty in monitoring and interpretation of bodily expressions

The patients described that their participation in healthcare planning was lacking and that the telemonitoring application did not contribute to a change in participation or support. They experienced difficulties in learning the technology and felt that the risk of deviating vital signs increased because the equipment was clumsy.

*I feel like there hasn't been any change between when I started and when I stopped, the values and everything have been where … at the same level the entire time, so I haven't had much use of it. (Patient 19)*

When technical problems arose due to, e.g., the telemonitoring application or measurement instruments not working as before, the patients felt stress and uncertainty. They described problems when synchronization between the measurement instruments and the application did not work, as well as difficulties in understanding the manual or getting the support they needed. The patients felt that the telemonitoring application was impersonal and did not include information adapted for each specific user. Also, they described a lack of information from healthcare professionals about specific, personalized self-care. For example, patients who had several different chronic illnesses would have multiple different treatments and self-care advice. The patients described uncertainty when the measured vital signs varied or when a change occurred. They also felt uncertain about if the healthcare professionals had received the vital signs, due to lacking communication. The patients were unsure about what needed to be done in case of worsening of their chronic illness with increasing symptoms or if vital signs deviated. This was framed as a lack of knowledge on how to maintain self-care activities. Further, uncertainty was described by the patients when self-care activities were performed but no results or effects were perceived.

## 4 Discussion

This study aimed to describe experiences of self-care management at home when living with hypertension or heart failure, with support from primary care through telemonitoring. The overarching theme, *'developing a capability to perform self-care with technology as both an intruder and an invited guest*', encompasses the latent meaning of the results. It suggests that the application used for supporting self-care can be viewed as an invited guest that facilitates 'development of expertise' in self-care management. This, in turn, helps patients and informal carers in ‘developing confidence’ to perform self-care management at home. Additionally, the application also promotes ‘a reciprocal relationship with healthcare professionals’, providing a sense of safety, independence, and support to both patients and informal carers. However, it was observed that not all patients with hypertension or heart failure felt at ease with the utilization of technical devices, nor did they develop the necessary skills to effectively manage their illness despite receiving technical support. This created a sense of alienation, accompanied by a dual perception of being both safe and unsafe simultaneously. They felt safe because they felt seen and supported through telemonitoring, but at the same time felt unsafe due to their incomplete understanding and limited ability to manage self-care monitoring. This fostered a sensation of intrusion, as if a stranger or intruder was encroaching upon their home.

The patients’ ambivalent experience of enhanced autonomy and self-care management, coupled with the sensation of insecurity and alienation when incorporating healthcare technology into their home, has also been demonstrated in other studies (Lindberg et al., 2021; Sundgren et al., 2020). As exemplified by Almevall et al. (2022), the home is central to the well-being of older adults. The familiarity of the home environment fosters a sense of control over daily life. A home is also an arena for social encounters, where daily activities are performed which also entails the self-care activities that are important to maintain health and independent living (Kylén et al., 2019). Living with a chronic illness like heart failure implies a gradual deterioration of health and functional ability, which may affect an older adult's sense of security and independence. Akerlind et al. (2018) revealed older patients' unease at night-time and apprehension about future living conditions and inadequate healthcare technology at home. Likewise, relatives' statements reflected concerns regarding the older adults’ health and limitations. Healthcare technology was perceived as re-establishing safety for both patients and their relatives. In line with our findings, the feeling of confidence was associated with someone keeping track of one's vital signs and the feeling of being unsafe was associated with technical malfunctions or a lack of technical competence. A sense of security in telemonitoring has been closely linked to the mutual engagement of healthcare professionals and patients, who collaborate in managing symptoms together (Ekstedt et al., 2023). This highlights the significance of empowering patients in self-care management and enhancing their digital competence when introducing telemonitoring at home. The integration of healthcare technology into the homes of older patients thus involves a complex interplay between enhanced autonomy and self-care management, alongside feelings of insecurity and alienation.

The findings showed how patients and informal carers gradually developed confidence in their self-care management by gaining real-life experience about how to adapt their self-care activities based on the vital signs. The study by Riegel et al. (2022b) revealed that monitoring of symptoms can create independence in one's own self-care management. Individuals who are uncertain about making self-care decisions often seek advice from healthcare professionals. The current study demonstrated challenges in maintaining healthy behaviours and engaging in self-care activities due to the deterioration associated with chronic illness. The difficulty in adapting self-care activities may arise from the progressive deterioration of the chronic illness, accompanied by the emergence of new symptoms over time. Our findings also indicated that the use of the telemonitoring application enhanced and developed patients' and informal carers' self-efficacy in self-care management. However, it is important to note that a study by Vidán et al. (2019) suggested patients with chronic illnesses may overestimate their self-care abilities. Our study also highlights the challenges associated with engaging patients with chronic illnesses in taking charge of their own care.

Our findings indicate the importance of acknowledging informal carers’ potential to motivate and encourage patients to sustain self-care management and overcome obstacles related to chronic illnesses. For instance, the involvement of informal carers in telemonitoring has been found to enhance patients' engagement in self-care monitoring of vital signs and improve symptom recognition by both informal carers and patients (Abdoli et al., 2019; Sabo and Chin, 2021). However, informal carers of persons with chronic illnesses often experience a significant burden and should get support for their own needs from healthcare professionals and be actively involved as partners in planning and information-sharing, together with the patient (Liljeroos et al., 2014; Whitehead et al., 2018). The healthcare professionals must get better at identifying needs and providing support to informal carers, as they bear a significant responsibility and caregiver burden for their loved ones, often without receiving adequate acknowledgement for their efforts (Nilsson et al., 2022).

This study also indicates that the digital tools offered a way to establish reciprocal relationships between patients, informal carers, and healthcare professionals. The opportunity to make individual adjustments in self-care activities after remote communication with healthcare professionals created a feeling of control over the chronic illness among the patients. In contrast, an absence of clearly defined goals and a lack of adequate follow-up by healthcare professionals resulted in a perceived lack of importance in sustaining self-care activities. Relatedness, i.e., feeling connected to others, is a core element that promotes motivation and engagement (Peters et al., 2018), confirming the importance of support from healthcare professionals and informal carers for sustained adherence to monitoring vital signs. According to Carlqvist et al. (2021), telemonitoring is perceived to create value when the tasks are aligned with a person's values and goals and the application adequately supports self-care or management of a chronic illness. According to the findings of this study, patients and informal carers can develop independence by establishing common goals together with healthcare professionals through the use of digital tools. Wildevuur et al. (2015) also emphasise the potential of eHealth solutions focusing on person-centred care to strengthen the use of shared goals and follow-ups.

The results of our study highlight the possibility of telemonitoring applications becoming a holistic eHealth innovation that enhances patients' well-being. There are still difficulties in developing holistic telemonitoring applications due to people's differing skills, social environments and undefined technical needs (Wannheden et al., 2021). To ensure person-centred care, it is essential to adopt a holistic approach that tailors telemonitoring to the specific needs and capabilities of each patient (Barello et al., 2016). However, implementing new services and processes in chronic care management requires navigating the complexities of varying patient needs and healthcare organizations, posing obstacles to a holistic approach (Ekstedt et al., 2022). Furthermore, the present study has highlighted the challenges involved in being in contact with healthcare professionals and the existence of multiple avenues for accessing healthcare services beyond the telemonitoring application. The use of a telemonitoring application in primary care may have the potential to evolve into a more accessible and easy way for persons with chronic illnesses to establish contact with healthcare professionals. This, in turn, indicates that the telemonitoring application can be perceived as a supportive tool or ‘an invited guest’ for home-based self-care, rather than as an intruder.

### 4.1 Implications

This study has shed light on the potential and challenges of telemonitoring to enhance high-quality care for individuals with hypertension or heart failure and their informal carers performing self-care activities at home. The use of telemonitoring in primary care offers a means of providing information and support between patients, informal carers and healthcare professionals, thereby enhancing the opportunity for increased self-care support. Telemonitoring in primary care may also reduce the risk of care exclusion for patients and informal carers. Telemonitoring applications have the potential to be part of healthcare services to reduce dependency and burdens for patients with chronic illness and their informal carers. Telemonitoring can be part of the process of learning how to adapt to a chronic illness and an opportunity for healthcare services to enhance interactions and support for patients with a chronic illness (Coster et al., 2020). The results of the current study showed that uncertainty in the self-care monitoring and interpretation of bodily expressions could still occur for patients with chronic illness. Healthcare professionals face challenges when taking on new roles and tasks that do not align with their existing workflows, particularly in providing support to patients with technology-related issues, for instance concerning telemonitoring applications or associated devices (Granja et al., 2018). Further education on the use of telemonitoring applications for healthcare professionals is needed, but integration into routines and working processes must also be performed (Granja et al., 2018; Li et al., 2021). However, realizing the full potential of any telemonitoring application requires further investigation to facilitate its integration into various healthcare settings, and to improve the accessibility of care for different age and patient groups. The findings in this study suggest that telemonitoring applications should have a dynamic nature and help patients and informal carers enhance their abilities to effectively manage and develop self-care capabilities.

### 4.2 Strengths and limitations

One strength of the study lies in its inclusion of both patients and informal carers, enabling a broad understanding of self-care management supported by telemonitoring applications. This could help future researchers highlight the importance of engaging informal carers in interventions aimed at enhancing patients' self-care.

Study limitations could arise from the constraints imposed by the pandemic, meaning that data collection was conducted through telephone interviews rather than the initially planned face-to-face interviews. Interpretation of the data may have been affected by the loss of visual cues. On the other hand, telephone interviews could encourage participants to speak more freely (Mealer and Jones, 2014; Novick, 2008). Conducting interviews over the telephone may also create a sense of ease and increased willingness among participants to disclose sensitive information (Mealer and Jones, 2014). When considering these potential advantages and challenges, telephone interviewing was deemed an effective method of data collection.

## 5 Conclusions

The findings in this study indicated that patients with hypertension or heart failure using telemonitoring applications at home could learn more about themselves and their illnesses. Even with the challenges that patients encounter in adapting to and accepting the day-to-day impact of progressive deterioration, healthcare professionals in primary care can offer support to both patients and informal carers through telemonitoring. Moreover, telemonitoring presents opportunities to enhance motivation and establish new routines and health goals in everyday life, fostering reciprocal relationships between patients and healthcare professionals.

## Funding

The Kamprad Family Foundation for Entrepreneurship, Research and Charity has financed this study through research grant no 20190249.

## Data statement

The raw interview data that were collected and analyzed in this manuscript are not publicly available due to participants not having consented to public availability. Aggregated data in Swedish are available from the corresponding author on reasonable request.

## CRediT authorship contribution statement

**Susanna Strandberg:** Conceptualization, Validation, Data curation, Formal analysis, Writing – original draft, Visualization. **Sofia Backåberg:** Conceptualization, Validation, Formal analysis, Writing – review & editing. **Cecilia Fagerström:** Conceptualization, Validation, Formal analysis, Writing – review & editing, Supervision, Funding acquisition. **Mirjam Ekstedt:** Conceptualization, Validation, Formal analysis, Writing – review & editing, Supervision, Project administration, Funding acquisition.

## Declaration of Competing Interest

None.

## Appendix Supplementary materials

Image, application 1
