# Supplementary material for: Self-care management and experiences of using telemonitoring as support when living with hypertension or heart failure: A descriptive qualitative study
Source: Int J Nurs Stud Adv. 2023 Aug 5;5:100149. doi: 10.1016/j.ijnsa.2023.100149 (PMC11080535; doi:10.1016/j.ijnsa.2023.100149)
Supplement: Supplementary file 1 [file mmc1.docx]

Appendices

Appendix A. Interview guide

- *Can you tell me about your experience of self-care management?*
- *Can you tell me what kind of self-care support you receive?*
- *Can you tell me about your experiences of using the telemonitoring application at home?*
- *Can you tell me about your experiences of self-care management with the telemonitoring application?*
- *Can you tell me what experiences you have of participation in healthcare when using a telemonitoring application?*
- *Can you tell me what sense of security you get from healthcare services?*
- *Do you want to add anything else on the topic before we end the interview?*

Follow-up questions:

- *Can you tell me more?*
- *Do you have any examples of that?*
- *What does that mean?*
- *How often or when?*
- *Who or what?*
